# A population-based study of rates of childbirth in recurrence-free female young adult survivors of Non-gynecologic malignancies

**DOI:** 10.1186/1471-2407-13-30

**Published:** 2013-01-23

**Authors:** Nancy N Baxter, Rinku Sutradhar, M Elizabeth DelGuidice, Shawn Forbes, Lawrence F Paszat, Andrew S Wilton, David Urbach, Linda Rabeneck

**Affiliations:** 1 Department of Surgery and Keenan Research Centre, Li Ka Shing Knowledge Institute, St Michael’s Hospital, University of Toronto, 30 Bond Street 16CC-40, Toronto, ON, M5B 1W8, Canada; 2 Institute for Clinical Evaluative Sciences, Toronto, Canada; 3 Institute of Health Policy, Management, and Evaluation, University of Toronto, Toronto, ON, Canada; 4 Department of Family and Community Medicine, University of Toronto, Toronto, Canada; 5 Department of Surgery, McMaster University, Hamilton, Canada; 6 Odette Cancer Centre, Sunnybrook Health Sciences Centre Toronto, Toronto, Canada; 7 Dalla Lana School of Public Health, University of Toronto, Toronto, Canada; 8 Department of Surgery, University Health Network, Toronto, ON, Canada; 9 Cancer Care Ontario, Toronto, Canada

**Keywords:** Cancer survivorship, Young adults, Pregnancy outcomes, Cohort study

## Abstract

**Background:**

Fertility is an important issue for long-term survivors of malignancies developing during reproductive years. We designed a population-based study to investigate childbirth in female young adult survivors of non-gynecologic malignancies.

**Methods:**

Women 20–34 years diagnosed with non-gynecologic malignancies in Ontario from 1992–1999 who lived at least 5 years recurrence-free were identified using the Ontario Cancer Registry and age matched to 5 randomly selected cancer-free women. Childbirth was determined through hospital discharge data. Time-to-childbirth was compared between survivors and controls using Cox proportional hazard regression for all subjects and stratified by prior childbirth and disease site.

**Results:**

3,285 survivors and 15,118 control women had a median of 12 years observation. 1,194 survivors and 6,049 controls experienced childbirth to the end of observation (March 2011). Overall, survivors experienced a longer time to childbirth than controls (HR 0.92, 95% CI 0.87-0.98), however this was limited to survivors with prediagnosis childbirth (HR 0.76, 95% CI 0.66-0.86). Survivors with no prediagnosis childbirth experienced a similar time to childbirth (HR 1.00, 95% CI 0.93-1.08) as control women. Differences between survivors and controls varied by type of malignancy; notably for those with prediagnosis childbirth, survivors of breast cancer (HR 0.45, 95% CI 0.29-0.68) and Hodgkin Disease (HR 0.57, 95% CI 0.36-0.91) had lower rates of postdiagnosis childbirth than controls.

**Conclusions:**

Long-term female young adult survivors of malignancies are less likely than controls to have childbirth after diagnosis; the overall effect is small and is influenced by prediagnosis childbirth and malignancy type.

## Background

Future fertility is important to many long-term survivors of malignancies that develop in the peak years of reproduction. With increasing numbers of women having children at later ages, even more cancer survivors will face this issue [1]. In recognition of the importance of future fertility, the American Society of Clinical Oncology published guidelines recommending discussion of the risk of infertility as a consequence of cancer treatment and referral for consideration of fertility preservation techniques when appropriate [2]. Although fertility is a common concern for young adult survivors (YAS) of malignancy and the clinicians caring for them, there are relatively few population-based studies addressing this issue in the literature. Studies from Finland [3] and Norway [4,5] demonstrate reduced fertility in the YAS population as compared to the general population or matched controls, however these studies include patients diagnosed over a long time period (as early as 1953 [3]) and reflect treatment regimens that have changed over time. Additionally, these studies include all patients who developed a malignancy at a young age including those with advanced disease and patients with rapid recurrence after treatment and thus may underestimate fertility in long-term survivors. Fertility of 5-year female survivors of childhood cancers has been evaluated as part of the Childhood Cancer Survivor Study [6]. Compared to sibling controls, the relative risk for female survivors ever being pregnant was 0.81 (95% confidence interval 0.73-0.90), but these findings have limited application to the YAS population. Other published studies include patients diagnosed with a single type of malignancy, and tend to be small, uncontrolled single institutions reports [7-10]. We therefore designed this study to evaluate childbirth in a population-based group of female young adult survivors of malignancy in Ontario Canada compared with matched control participants without a cancer diagnosis.

## Methods

We designed a retrospective, population-based cohort study using a provincial cancer registry linked to administrative data sets.

### Data sources

We used four data sources:

1. The Ontario Cancer Registry (OCR) includes information on all incident cancers diagnosed since 1964 in Ontario. Reporting is provincially mandated and over 95% complete [11]. The OCR does not maintain information on tumor stage and does not contain treatment information.

2. The Ontario Health Insurance Plan (OHIP) database contains information on claims billed by physicians for services, permitting identification of virtually all medical procedures occurring in Ontario.

3. The Canadian Institute for Health Information Discharge Abstract Database (CIHI-DAD), contains information on every patient discharged from a hospital or same-day surgery unit in Ontario and is highly accurate for admissions for pregnancy and childbirth [12].

4. The Registered Persons Database (RPBB) is a roster of all OHIP beneficiaries (virtually all individuals living in Ontario) and includes demographic information and length of eligibility.

Diagnostic and procedure codes used in this study are presented in Additional file 1.

### Selection of survivors

Female YAS were identified using the OCR. While the definition of a YAS varies [13], we limited our cohort to women age 20 through 34 at diagnosis. All female young adults registered in the OCR between 1992 (when datasets became reliably linkable) and 1999 (to enable substantive follow up for all 5 year survivors) were eligible for inclusion. Women were excluded if they died within five years of diagnosis, were diagnosed with a gynaecological malignancy, were registered in OCR for a previous malignancy, or were not continuously eligible for provincial health insurance coverage for at least seven years after diagnosis (or until death).

### Identification of recurrence

Recurrence of malignancy is likely to have an influence on childbearing but the OCR does not include information on cancer recurrence. We therefore developed an algorithm to identify survivors with evidence of recurrent disease from physician claims and diagnostic codes based on use of chemotherapy, palliative care, or diagnosis of metastatic disease (Additional file 1). Patients with a solid tumor malignancy undergoing a second course of chemotherapy (or third course in the case of hematologic malignancies) after completion of adjuvant therapy, or delivery of a first course of chemotherapy more than 6 months after completion of cancer directed surgery were considered to have disease recurrence. Survivors identified with recurrent disease within the first 5 years of diagnosis were excluded entirely. Survivors developing recurrent disease after 5 years of survivorship were censored 6 months before the date recurrence was identified as the exact date of recurrence could not be obtained.

### Selection of controls

A female control population was selected using the RPDB. Eligible women from the general population were matched to the survivors based on calendar year of birth and geographic location. Five controls were randomly selected without replacement from all potential controls matched to a given survivor. Controls were assigned a referent date that corresponded to the date of diagnosis in the matched survivor. Controls were excluded if they had a diagnosis of cancer prior to the referent date (determined through linkage with the OCR), died within five years of the referent date, or were not continuously eligible for provincial health insurance for at least seven years after the referent date.

### Identification of surgical sterilization

We identified women who had undergone a procedure consistent with surgical sterilization (tubal ligation, bilateral oophorectomy, hysterectomy) based on OHIP and CIHI-DAD codes. Survivors and controls with evidence of surgical sterilization at any time prior to diagnosis or up to 12 months after diagnosis or referent date were excluded. Individuals undergoing surgical sterilization more than 1 year after diagnosis or referent date were censored on the date of surgical sterilization.

### Determining childbirth

We identified admission for childbirth for all members of our cohort from Jan 1, 1987 through March 31, 2011 from information from CIHI-DAD. Delivery of an infant, live or stillborn over 20 weeks gestational age, as coded in CIHI was considered evidence of childbirth for this study.

### Covariates

For survivors and controls we determined income quintile, defined by the census dissemination area where individuals lived at the date of diagnosis or referent date. We considered childbirth prior to the date of diagnosis or referent date a potential covariate. For the survivor group we evaluated rates of delivery by diagnosis, categorizing survivors into broad groups including the diagnoses with at least 100 women (brain, breast, Hodgkin lymphoma, non-hodgkin lymphoma [NHL], melanoma, thyroid and other malignancies).

### Analysis

We calculated descriptive statistics for study variables stratified for survivors and controls. The outcome of interest was childbirth occurring at least one year after the date of diagnosis (survivors) / referent date (controls). The one year interval was used to ensure that childbirth was a result of post-diagnosis pregnancy. We calculated the time between diagnosis or referent date to the time of admission to hospital for childbirth for each subject. Patients were censored at death, loss-to-follow-up, surgical sterilization, 6 months prior to evidence of recurrent disease, or March 31, 2011, whichever came first. Multivariate analyses with the Cox proportional hazards regression model were conducted to evaluate the relationship between time to childbirth and covariates such as YAS (yes or no), income quintile, age (treated as continuous), and previous childbirth (children born prior to diagnosis or referent date, yes or no). did not consider childbirth from 0–12 months from diagnosis/referent date in our analysis. We tested the interaction between the survivor indicator and previous childbirth. Since this interaction term was highly significant, we further matched survivors and corresponding controls on previous childbirth and stratified the analysis based on this variable. That is, the first and second stratum consists of all survivors and corresponding matched controls with and without, respectively, children born prior to diagnosis or referent date. The Cox regression model for each stratum included YAS, income quintile, and age. To account for the matched design with a variable number of controls per survivor (due to further matching by previous childbirth), we used a robust sandwich variance estimator approach to estimate the standard errors of the Cox regression parameter estimates [14]. The proportional hazards assumption was tested and was not violated. We repeated the analysis without censoring patients for recurrence after 5 years as a sensitivity analysis.

We analyzed data using SAS version 9.2 (Cary, North Carolina). All statistical tests were two-sided, and p-values less than 0.05 were considered statistically significant. The study was approved by the Research Ethics Board of St. Michael’s Hospital, Toronto, Ontario. All data analysis was conducted at the Institute for Clinical Evaluative Sciences, a Section 45 (1) prescribed entity in Ontario’s Personal Health Information Protection Act. The data used are not freely accessible; permission for the use of the data was given by the Institute for Clinical Evaluative Sciences.

## Results

We identified 5,172 women age 20 through 34 who developed a non-gynecologic invasive malignancy between Jan 1, 1992 and Dec 31, 1999 based on registration in the OCR. Of these, 3,536 survived at least 5 years after diagnosis with no evidence of recurrence in administrative data and were continuously eligible for health insurance in Ontario until death or at least 7 years after diagnosis. There were 3,2 85 YAS after all exclusion criteria were applied (consort diagram) Additional file 2. We selected 15,176 matched controls from a potential control population of 2,660,134 women. The characteristics of the survivors and controls are presented in Table 1. The majority of survivors had breast cancer (18%), thyroid cancer (27%) or melanoma (15%) (Table 2).

**Table 1:** Characteristics of female young adult survivors and their matched controls

	**All**		**Prediagnosis Childbirth**		**No Prediagnosis Childbirth**	
	**Young Survivors (N = 3,285)**	**Controls (N = 15,176)**	**Young Survivors (N = 1,093)**	**Controls (N = 2,066)**	**Young Survivors (N = 2,192)**	**Controls (N = 6,937)**
Age, Mean (SD)	28.8 (4.1)	28.6 (4.1)	30.1 (3.3)	30.8 (2.8)	28.1 (4.3)	27.3 (4.4)
Median Follow-up in survivors without childbirth	12.4	13.0	11.7	11.9	12.8	13.6
Diagnosis Year (%)						
1992	397 (12.1)		92 (8.3)		305 (13.9)	
1993	387 (11.8)		109 (10.0)		278 (12.7)	
1994	386 (11.8)		110 (10.0)		276 (12.6)	
1995	400 (12.2)		142 (12.4)		258 (11.8)	
1996	385 (11.7)		134 (12.3)		251 (11.4)	
1997	410 (12.5)		150 (13.8)		260 (11.9)	
1998	441 (13.4)		177 (16.3)		264 (12.0)	
1999	479 (14.6)		179 (17.0)		300 (13.7)	
Income Quintile (%)						
1 (lowest)	662 (20.2)	3138 (20.7)	210 (19.2)	395 (19.1)	452 (20.6)	1413 (20.4)
2	677 (20.6)	3269 (21.5)	214 (19.6)	425 (20.6)	463 (21.1)	1502 (21.7)
3	644 (19.6)	3066 (20.2)	218 (20.0)	423 (20.5)	426 (19.4)	1381 (19.9)
4	681 (20.7)	2975 (19.6)	246 (22.5)	465 (22.5)	435 (19.8)	1315 (19.0)
5 (highest)	621 (18.9)	2728 (18.0)	205 (18.8)	358 (17.3)	416 (19.0)	1326 (19.1)
Surgical Sterilization 12 months or more after Diagnosis or Referent Date (%)						
Yes	637 (19.4)	2,609 (17.2)	305 (27.9)	520 (25.2)	332 (15.1)	855 (12.3)
No	2,648 (80.6)	12,567 (82.8)	788 (72.1)	1,546 (74.8)	1,860 (84.9)	6,082 (87.7)
Childbirth prior to diagnosis or Referent Date (%)						
Yes	1,093 (33.3)	5,342 (35.2)	1,093 (100)	2,066 (100)		
No	2,192 (66.7)	9,834 (64.8)			2,192 (100)	6,937 (100)
Childbirth 12 months or more after Diagnosis or Referent Date (%)						
Yes	1,194 (36.3)	6,049 (39.9)	336 (30.7)	716 (34.7)	858 (39.1)	2,983 (43.0)
No	2,091 (63.7)	9,127 (60.1)	757 (69.3)	1,350 (65.3)	1,334 (60.9)	3,954 (57.0)
Mean number of deliveries						
12 Months or more post diagnosis/referent date	0.58	0.63	0.39	0.43	0.68	0.76
At any time pre or post diagnosis/referent date	1.05	1.12	1.80	1.87	0.68	0.76
Cumulative 10-year delivery rate	36.3	39.1	32.5	36.2	38.0	40.4

**Table 2:** Rates of childbirth 12 months after diagnosis / referent date over time

**Type of Malignancy**	**N (% of total)**	**% Childbirth Prediagnosis**	**Mean # Postdiagnosis Deliveries**		**Cumulative 10-year Rate of Childbirth (%)**	
			**Survivors**	**Controls**	**Survivors**	**Controls**
Brain	142 (4.3)	35.9	0.42	0.72	31.2	41.6
Breast	588 (17.9)	34.5	0.30	0.43	22.5	29.9
Hodgkin Lymphoma	358 (10.9)	22.9	0.82	0.81	43.2	46.4
Non-Hodgkin Lymphoma	204 (6.2)	22.6	0.53	0.60	34.6	37.6
Thyroid	890 (27.1)	36.7	0.67	0.67	40.8	41.4
Melanoma	498 (15.2)	36.1	0.69	0.63	41.1	39.9
Other	605 (18.4)	33.7	0.57	0.64	35.6	38.9

**Figure 1:**
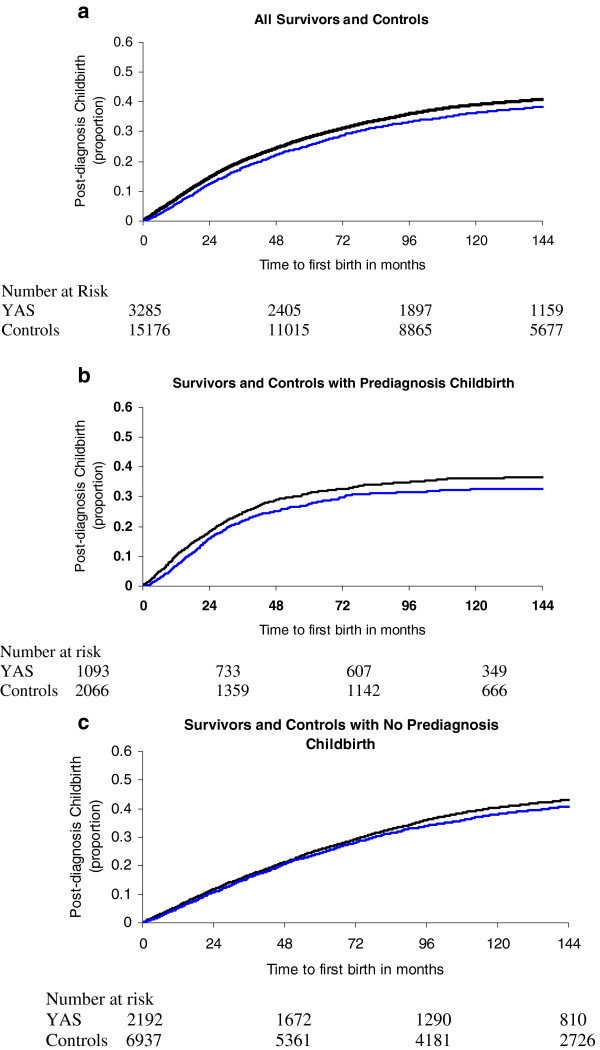
**Time to childbirth at least 12 months post diagnosis / referent date.** The blue line represents the YAS and the black line represents the control group.

A total of 1,194 of survivors delivered 1,910 children in the period from 1 year after diagnosis to the end of follow up vs. 6,049 controls who delivered 9,516 children. Survivors in our cohort were less likely than controls to be admitted for childbirth starting 12 months or more after diagnosis (Figure 1); the cumulative rate of childbirth at 10-years in the survivor group was 36.3% vs. 39.9% in the control group (p < 0.001). After adjusting for socioeconomic status and age in our multivariate model, time to childbirth was significantly longer for survivors than controls (HR 0.92, 95% CI 0.87–0.98) (Table 3). Childbirth prior to diagnosis influenced time to childbirth after diagnosis (Figure 1); to further evaluate this relationship we stratified our survivors by known childbirth prior to diagnosis and included only controls with a similar history prior to the referent date. There were 1,093 survivors and 2,066 matched controls with childbirth prior to the diagnosis/referent date and 2,192 survivors and 6,937 matched controls without childbirth prior to the diagnosis/referent date. As compared to controls, survivors with prior childbirth were less likely to experience a delivery over time (HR 0.76, 95% CI 0.66–0.86) while survivors without prior childbirth had a similar rate of childbirth (HR 1.00, 95% CI 0.92–1.08) (Table 3). The results did not change when we did not censor 5-year survivors 6 months prior to evidence of recurrence.

**Table 3:** Results of multivariable cox proportional hazards model evaluating time to childbirth more than 1 year after diagnosis/referent date by survivor status

**Variable**	**Overall**			**Women with childbirth before diagnosis**			**Women with no childbirth before diagnosis**		
	**HR**	**95% CI**	**p value**	**HR**	**95% CI**	**p value**	**HR**	**95% CI**	**p value**
Survivor Status									
YAS	0.92	0.87–0.98	0.007	0.76	0.66–0.86	<0.001	1.00	0.93–1.08	0.93
Control	1	Referent		1	Referent		1	Referent	
Income Quintile									
1	0.89	0.83–0.95	0.001	0.70	0.57–0.86	<0.001	0.85	0.77–0.94	0.002
2	0.92	0.86–0.99	0.02	0.72	0.59–0.89	0.002	0.94	0.86–1.04	0.23
3	0.94	0.87–1.01	0.08	0.84	0.69–1.02	0.09	0.90	0.82–0.99	0.04
4	1.00	0.93–1.07	0.95	0.86	0.70–1.04	0.12	1.01	0.92–1.11	0.89
5	1	Referent		1	Referent		1	Referent	
Age	0.91	0.90–0.91	<0.001	0.86	0.85–0.88	<0.001	0.91	0.91–0.92	<0.001

**Figure 2:**
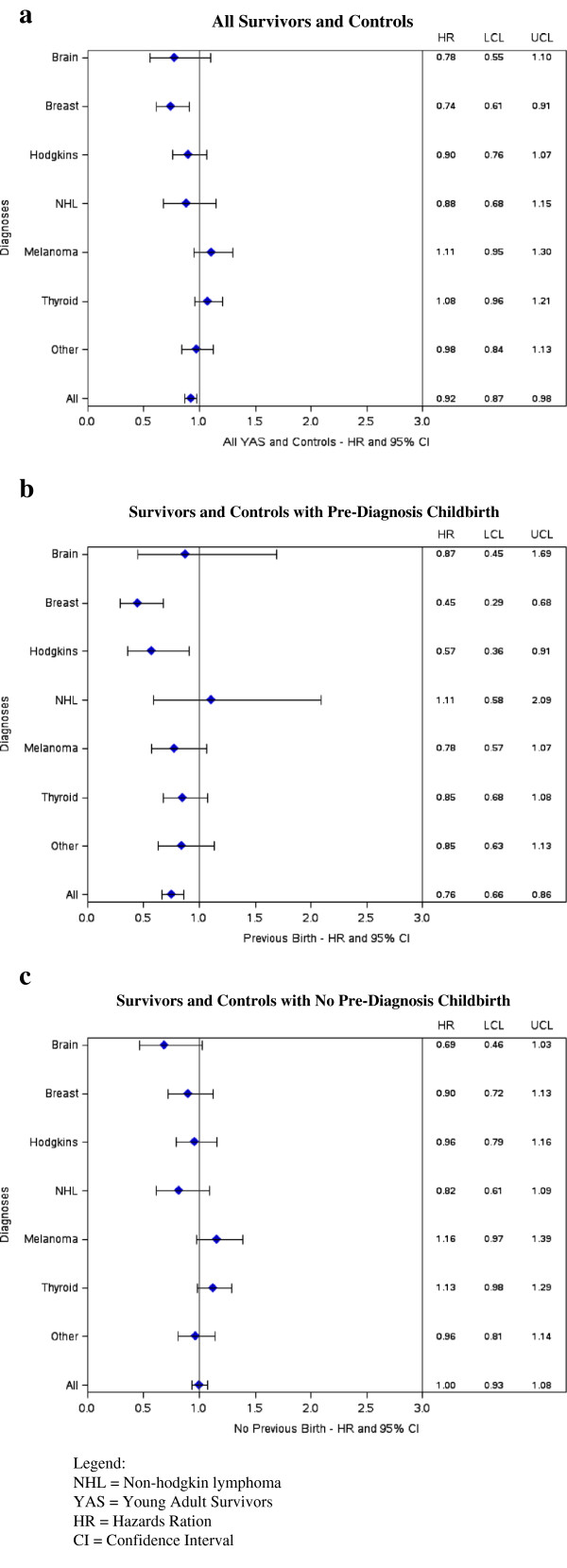
**Results of multivariable cox proportional hazards model evaluating time to childbirth at least 12 months after diagnosis / referent date by survivor status for individual malignancies controlling for age and SES.** Figure 2b: Survivors and Controls with Pre-Diagnosis Childbirth. Figure 2c: Survivors and Controls with No Pre-Diagnosis Childbirth.

The relationship between time to childbirth and survivor status varied by diagnosis (Table 3, Figure 2); notably for those with prior childbirth, YAS with breast cancer (HR 0.45, 95% CI 0.29–0.68) or Hodgkin disease (HR 0.57, 95% CI 0.36–0.91) were statistically significantly less likely to experience childbirth than controls. In contrast, a cancer diagnosis had no statistically significant impact for other YAS groups. Notably, women with thyroid cancer and melanoma experienced very similar rates of childbirth as control women (cumulative 10-year rate of child birth in YAS with thyroid cancer 40.8% vs. controls 41.4% and in YAS with melanoma 41.1% vs. 39.9%)

## Discussion

In this population based study of young female survivors of non-gynecological malignancy, time to childbirth was greater than age-matched women with no history of malignancy; as compared to matched controls over a median observation time period of 12 years after diagnosis although the difference was small (HR 0.92, 95% CI 0.87–0.98). Notably, the relationship between childbirth and survivor status varied significantly by history of previous childbirth; survivors with no history of previous childbirth actually had a similar likelihood of postdiagnosis childbirth as matched controls, while for those with children postdiagnosis childbirth was reduced by 24%.

There are 3 previously published population-based studies of pregnancy and parenthood after a diagnosis of any malignancy in young adults [3-5]; all included patients diagnosed from the 1960s. Only one study, conducted in Norway [5], included all survivors irrespective of parenthood status before diagnosis and found, similar to our study, a significant relationship between previous pregnancy and the rate of postdiagnosis pregnancy – overall the rate of pregnancy was lower in female cancer patients than controls (HR 0.61) however this was more pronounced for women with a child prior to diagnosis (HR 0.52) than for those without a child (HR 0.73). A large cohort study of young adults treated at a single institution in Norway [15,16] demonstrated similar findings. Variations in attitudes towards parenthood may be the cause of this finding. Perceptions of young adult cancer survivors with respect to fertility has been found to vary with parenthood status; childless survivors have been found more likely to desire future children and are less likely to perceive that the diagnosis of cancer has negatively influenced their desire for future children than survivors with children [17].

The two additional population-based studies [3,4] evaluated first time parenthood (i.e. deliveries in survivors with no children prior to diagnosis) in survivors. Syse et al [4] demonstrated a 27% reduction in parenthood in women diagnosed with cancer between age 17–44 as compared to the population while Madanat [3] demonstrated a 54% reduction in parenthood in women diagnosed with cancer between age 0–34 as compared to sibling controls. These studies demonstrated a greater impact of cancer diagnosis on postdiagnosis pregnancy or parenthood than ours and this may be explained in a number of ways. The studies were not limited to patients surviving cancer, and they therefore included individuals with advanced disease at diagnosis, those with limited life expectancy and patients who developed recurrence within a short period of follow up. Such patients would be expected to have a lower rate of pregnancy than long-term survivors. Additionally, these studies included patients diagnosed over a long period of time – all found higher rates of pregnancy in young adult patients treated in more contemporaneous time periods, although follow up of patients in the later time periods in these studies was limited. Finally, we did not include patients with gynaecologic malignancies, a group most likely to undergo surgical sterilization as part of treatment.

Type of malignancy was associated with obstetrical delivery post-diagnosis. Breast cancer survivors who had a history of prior childbirth had a marked reduction in the rate of postdiagnosis childbirth (HR 0.45, 95% CI 0.29–0.68) as compared with controls. There are a number of factors that may influence the fertility of women after a diagnosis of breast cancer [18,19]. Many young women who develop breast cancer receive chemotherapy to improve their chance of survival but are therefore at risk of chemotherapy-related amenorrhea, premature ovarian failure and infertility. Exposure to prolonged hormonal therapy in women with ER positive breast cancer reduces fertility for the duration of exposure. However, given that women with breast cancer with no prediagnosis childbirth had similar rates of childbirth to controls (HR 0.90, 95% HR 0.72–1.13) decreased fertility in these patients is likely an incomplete explanation of our finding. Although studies have not demonstrated a negative impact of pregnancy on breast cancer outcome [20,21] it is possible that women may delay or avoid pregnancy for fear of recurrence with the estrogen stimulation of pregnancy; this effect may be more pronounced in women who have had children. Survivors of Hodgkin Disease who had prior childbirth were similarly less likely to experience postdiagnosis deliveries (0.57, 95% CI 0.36–0.91) than control women. Patients with Hodgkin Disease are frequently exposed to oophorotoxic chemotherapy and premature ovarian failure is common after bone marrow transplantation [22]. However, the cumulative 10-year rate of childbirth in YAS with Hodgkin Disease was substantial (Hodgkins 43.2%) and only 3.2% lower than matched controls.

This is a large population-based study and all patients included were treated since 1990 with therapeutic interventions more likely to be relevant to today’s survivors than many previous studies. Additionally, by limiting our sample to 5 year survivors we excluded most women with advanced disease, limited life expectancies and early recurrence, groups with expected lower rates of childbirth. Inclusion of these women would tend to overestimate the impact of a cancer diagnosis on childbirth for long-term survivors. Our study, with observation to March 2011 provides over 10 years of potential follow up to all women included in the cohort. Our study however does have limitations. To ensure a contemporary cohort of 5-year survivors with sufficient follow up we have restricted our cohort to an 8 year period and therefore the sample size for some types of malignancies is small. Additionally, information regarding childbirth is available only since 1988 and thus we do not have complete prediagnosis information for all women. We do not have access to data on assisted reproduction as this was not a covered service in Ontario and thus we do not know if YAS required such services more frequently than controls. The study may be influenced by factors specific to a Canadian context and factors such as underlying fertility of the population [23] should be considered prior to generalizing to other jurisdictions. Finally, we do not have access to detailed information regarding stage or treatment which would further inform the analysis.

## Conclusion

Female survivors of malignancy developing in young adulthood overall experience a small reduction in the likelihood of childbirth after diagnosis over a median of 12 years of follow up as compared to age matched controls, however the effect is not homogeneous. Parenthood prior to diagnosis modifies the effect of diagnosis – women who did not have children prior to diagnosis were more likely to experience childbirth after diagnosis indicating that non-biologic factors have an important influence in this group. Survivors of melanoma and thyroid cancer, particularly those with no history of childbirth, can be reassured by our findings that for long term survivors reproductive outcomes do not seem to be affected. However, for women surviving other forms of malignancies, in particular breast cancer and Hodgkin Disease there is a reduction in childbirth even in this comparatively contemporary cohort. These findings should be considered in pre-treatment counselling and discussions with patients with respect to options to preserve fertility in those desiring future pregnancies. However, given the association between prediagnosis childbirth and the likelihood of childbirth after diagnosis, non-biologic factors may have an important influence on the likelihood of having children after a malignant diagnosis in the YAS population.

### Research support

This research was supported by the Canadian Institutes for Health Research and the Ontario Ministry of Research and Innovation. Dr Baxter holds the Cancer Care Ontario Health Services Research Chair and an Early Researchers Award from the Ontario Ministry of Research and Innovation. Dr. Paszat is supported by a clinician scientist salary from the Ministry of Health and Long-term Care of Ontario. The funding sources played no role in design, conduct, or reporting of this study. This study was supported by the Institute for Clinical Evaluative Sciences, which is funded by an annual grant from the Ontario Ministry of Health and Long-Term Care. The opinions, results and conclusions reported in this paper are those of the authors and are independent from the funding sources. No endorsement by the Institute for Clinical Evaluative Sciences or the Ontario Ministry of Health and Long-Term Care is intended or should be inferred.

## Competing interests

The authors declare that they have no competing interests.

## Authors’ contribution

NNB was responsible for study design, analysis and drafting of the manuscript. RS participated in study design, was responsible for the analysis and helped to draft the manuscript. EG participated in study design and helped to draft the manuscript. SF participated in study design and analysis. FLP participated in study design, analysis and edited the manuscript. ASW performed the analysis and helped to draft the manuscript. DU participated in study design and analysis. LR participated in study design, analysis and edited the manuscript. All authors read and approved the final manuscript.

## Pre-publication history

The pre-publication history for this paper can be accessed here:

http://www.biomedcentral.com/1471-2407/13/30/prepub

## Supplementary Material

Additional file 1: Appendix 1 Diagnostic and Procedure Codes used in the Study. Click here for file

Additional file 2: Appendix 2 Consort Diagram. Click here for file
