# Supplementary material for: A population-based study of rates of childbirth in recurrence-free female young adult survivors of Non-gynecologic malignancies
Source: BMC Cancer. 2013 Jan 23;13:30. doi: 10.1186/1471-2407-13-30 (PMC3605316; doi:10.1186/1471-2407-13-30)
Supplement: Additional file 1: Appendix 1 — Diagnostic and Procedure Codes used in the Study. [file 1471-2407-13-30-S1.doc]

Appendix 1: Diagnostic and Procedure Codes used in the Study

| OCR | ICD-9 codes |
| --- | --- |
| Non-gynecologic malignancies | 140-149, 160, 161: Head And Neck  153, 154: Colorectal (excluding 153.5, 154.2, 154.3, 154.8)  172: Melanoma  174: Breast  188, 189: Urologic  191, 192: Brain  193: Thyroid  200, 202: NHL  201: Hodgkin Lymphoma  204-208: Leukemia  OTHER: Other |
| Gynecologic malignancies | 179, 180, 182, 183 |
| CIHI-DAD | Diagnostic Codes: ICD-9 codes (1988-2001), ICD-10 codes (2002-2010)  Procedure Codes: CCP codes (1988-2001), CCI code (2002-2010) |
| Metastatic Disease | ICD-9: 1960, 1961, 1962, 1965, 1968, 1969, 1970, 1971, 1972, 1973, 1974, 1975, 1976, 1977, 1978, 1980, 1981, 1982, 1983, 1984, 1985, 1986, 1987, 1988, 1990, 1991  ICD-10: C770, C771, C772, C774, C775, C778, C779, C780, C781, C782, C783, C784, C785, C786, C787, C788, C790, C791, C792, C793, C794, C795, C796, C797, C798 |
| Bone Marrow Transplant | CCP: 530, CCI: 1WY19 |
| Obstetrical Delivery | 1988-2001: Patient service (PATSERV)=Obstetrical Delivered (51), OR Case Mix group (CMG) in 599, 600, 606, 607, 608, 609, 610, 611, 601, 602, 603, or 604  2002: : PATSERV=51, OR CMG in 599, 600, 606, 607, 608, 609, 610, 611, 601, 602, 603, or 604, OR any CCI code (INCODE) in 5.MD.50.xx-5.MD.60.xx, OR sixth digit for O00-O99 is 1 or 2, OR ICD-10 (DX10CODE) codes Z370-Z73791 with diagnosis type (DXTYPE)= M, 1, 2, W, X, or Y  2003-2010: PATSERV=51, OR CMG in 536-545, OR any INCODE in 5.MD.50.xx-5.MD.60.xx, OR sixth digit for O00-O99 is 1 or 2, OR ICD-10 codes Z370-Z73791 with DXTYPE M, 1, 2, W, X, or Y |
| Surgical Sterilization | Fallopian Tube Excision/Destruction  CCI: 1RF51, 1RF59, 1RF87, 1RF89, CCP: 78.2, 78.3, 78.4, 78.53  Hysterectomy  CCI: 1RM57, 1RM59, 1RM89, 1RM91, CCP: 80.2-80.7  Oophorectomy  CCI: ??, CCP: 77.4 |
| OHIP Feecode | OHIP schedule of benefits |
| Metastatic Disease | Chemotherapy: G381, G281, G339, G345, G359, G390  Palliative Care: A945, C945, W982, C982, W882, C882, W872, W972, K023 |
| Surgical Sterilization | Fallopian Tube Excision/Destruction: S741  Hysterectomy: S757, S816, S758, S759, S710, S763  Oophorectomy: Unilateral and bilateral oophorectomy cannot be distinguished. OHIP codes for oophorectomy therefore not used |
