# Supplementary material for: A population-based study of rates of childbirth in recurrence-free female young adult survivors of Non-gynecologic malignancies
Source: BMC Cancer. 2013 Jan 23;13:30. doi: 10.1186/1471-2407-13-30 (PMC3605316; doi:10.1186/1471-2407-13-30)
Supplement: Additional file 2: Appendix 2 — Consort Diagram. [file 1471-2407-13-30-S2.doc]

Appendix 2

**Selection of Survivors**

All women aged 20-34 with non-gynecologic malignancy in OCR diagnosis date 1992-1999 (n=5,200)

Include only First Cancer Diagnosis (excludes 34)

Include only those who survived at least 5 years

(excludes 841)

Include only those with at least 2 years of provincial insurance coverage after year 5 or continuous coverage to death (excludes 417)

Include only those with no evidence of recurrence before 5 years of survivorship (excludes 372)

Include only those with no evidence of surgical sterilization before 5 years of survivorship (excludes 251)

**Total # survivors = 3,285**
